# Editorial: NK Cell Subsets in Health and Disease: New Developments

**DOI:** 10.3389/fimmu.2017.01363

**Published:** 2017-10-18

**Authors:** Emanuela Marcenaro, Luigi D. Notarangelo, Jordan S. Orange, Eric Vivier

**Affiliations:** ^1^Department of Experimental Medicine (DIMES), Center of Excellence for Biomedical Research (CEBR), University of Genova, Genova, Italy; ^2^Immune Deficiency Genetics Section, Laboratory of Clinical Immunology and Microbiology, National Institute of Allergy and Infectious Diseases, National Institutes of Health, Bethesda, MD, United States; ^3^Department of Pediatrics, Baylor College of Medicine, Center for Human Immunobiology, Texas Children’s Hospital, Houston, TX, United States; ^4^Centre d’Immunologie de Marseille-Luminy, Aix Marseille Université, INSERM, CNRS, Marseille, France; ^5^Service d’Immunologie, Hôpital de la Timone, Assistance Publique-Hôpitaux de Marseille, Marseille, France

**Keywords:** natural killer cell subsets, immune checkpoints, innate lymphoid cells, anti-tumor responses, anti-viral responses, immunotherapy

**Editorial on the Research Topic**

**NK Cell Subsets in Health and Disease: New Developments**

Natural killer (NK) cells were discovered *ca* 1975, as the first group of lymphoid cells that were neither T cells nor B cells. Since then, the dissection of the biology of NK cells has been growing exponentially with many seminal discoveries from the identification of MHC class I-specific inhibitory receptors to the discovery of receptor–ligand pairs involved in NK cell activation and to the manipulation of NK cells in cancer.

In this research topic, we asked a group of thought leaders in NK cell biology to review recent advances in their origins and biology, and their roles in cancer, infection, and inflammation.

Together, these 25 articles provide a timely survey of NK cells as critical immunologic components of health and disease. They will hopefully prompt further dialog and developments in basic and translational immunology.

## NK Cell Origins and Biology

Nowadays, NK cells are recognized to belong to the family of innate lymphoid cells (ILCs) that include other subsets of lymphoid cells, such as Lymphoid Tissue-inducer cells (LTi), ILC1, ILC2, and ILC3 (Jiao et al.). Human NK cells normally constitute 5–15% of human peripheral blood (PB) lymphocytes. Human PB NK cells can be distinguished in two subsets according to their surface expression of CD56: CD56^bright^ and CD56^dim^. CD56^bright^ human NK cells express high levels of CD94/NKG2A but low levels of CD16 and lack of KIRs; they predominate in lymph nodes and express low baseline levels of perforin and cytotoxic activity. In addition they produce high levels of cytokines, including IFN-γ, following stimulation by pro-inflammatory cytokines. On the other hand, CD56^dim^ NK cells are CD16^high^ and express KIRs and/or CD94/NKG2A; they predominate in PB (about 90% of PB NK cells); they show high baseline levels of perforin and cytotoxicity against tumor/virus-infected target cells; and they also produce various cytokines in response to direct target cell interactions (Scoville et al.). A third NK cell subset is represented by the CD16^+^CD56^neg^ NK cells, that are poorly functional, express low levels of natural cytotoxicity receptors (NCRs) and Siglec-7 (an inhibitory lectin-type receptor expressed on the majority of NK cells) and may become more abundant in PB during persistent inflammation, characterizing several human chronic immunological diseases (such as viral infections and autoimmune diseases) (Mikulak et al.).

It has been recently demonstrated that the developmental pathways for NK cell and ILC development are distinct; however, the developmental relationship between the different types of human NK subsets has not been finally clarified. While long held to represent sequential linear stages in maturation, more recent hypotheses propose that CD56^bright^ NK cells may represent NK cells activated *in vivo* and/or that PB NK cell subpopulations derive from different hematopoietic progenitor cells. In this context, a recent study using genetic bar coding of NK cell lineages in rhesus macaques was suggestive of the two subsets having distinct ontologies (Scoville et al.). In addition, the recent characterization of a novel CD34^+^DNAM-1^bright^CXCR4^+^ precursor in PB of patients with chronic inflammatory conditions giving rise to apparently licensed and functional maturing NK cells may suggest the possibility for a higher than expected common lymphocyte precursor diversity and a consequently higher peripheral NK cell phenotype variability (Bozzano et al.).

Despite the existence of alternative hypotheses for NK cell development, continually accumulating evidence suggest that a least fraction of CD56^bright^ NK cells represent direct physiologic precursors of CD56^dim^ NK cells. During their process of differentiation, CD56^dim^ NK cells lose expression of CD94/NKG2A and subsequentially acquire inhibitory KIRs and LIR-1, in addition they upregulate T-bet and downregulate Eomes, two key transcription factors regulating NK cell maturation and function during the last steps of their differentiation. In this context, it has been demonstrated that although the number of KIRs correlates with the extent of T-bet/Eomes modulation, “the modulation of these T-box transcription factors during NK cell maturation does not depend on signals conveyed by KIR” (Pradier et al.; Simonetta et al.). The terminally differentiated phenotype of CD56^dim^ cells is marked by the expression of the CD57 molecule that is associated with poor ability to respond to cytokine-mediated stimulation, but preserved cytolytic capacity (Scoville et al.; Bozzano et al.; Della Chiesa et al.).

Notably, within the total CD56^bright^ NK cell subset, a phenotypic and functional heterogeneity can be observed, as shown by the existence of tissue-resident CD56^bright^ NK cells in the uterus/maternal decidual, liver, and lymphoid tissues, where these cells exert tissue-specific functions. Uterine CD56^bright^ NK cells express CD49a while the liver- and lymphoid tissue-resident CD56^bright^ NK cells co-express CD69 and CXCR6. There is increasing evidence that decidual NK (dNK) cells are crucial for pregnancy. Recent data demonstrate that the functions of dNK cells could be suppressed by a reduced HLA-G expression on extravillous trophoblasts suggesting a possible mechanism of recurrent miscarriage (Melsen et al.; Guo et al.). Finally, CD56^bright^ NK cells may also play a major role in controlling T cell responses and maintaining homeostasis. In this context, disorder in the regulation function of NK cells has been recently described in patients with untreated multiple sclerosis (MS), indicating a possible role of this subset in the MS pathogenesis (Gross et al.).

## NK Cells and Viruses

In recent years, it has been appreciated how infection with human cytomegalovirus (HCMV) can shape the NK cell receptor repertoire by inducing an increased frequency of a CD57^+^ NK cell population expressing CD94/NKG2C, the activating counterpart of CD94/NKG2A. This HCMV-induced NK cell subset is characterized by the CD56^dim^CD16^bright^LIR-1^+^KIR^+^NKG2A^−^ surface phenotype and expresses KIRs interacting with self-HLA class I molecules. In addition, these NK cells can display some hallmarks of adaptive immunity, such as, enhanced effector function, longevity, clonal expansion as well as given epigenetic modifications, including epigenetic remodeling at the *IFNG* locus, decreased expression of certain signaling molecules (i.e., the adaptor protein FcRγ and the tyrosine kinase Syk), and lower expression levels of the transcription factor PLZF. The higher accessibility of the *IFNG* locus can increase IFN-γ production upon target stimulation, while the lack of FcRγ can induce killing *via* antibody-dependent cellular cytotoxicity (ADCC) of opsonized HCMV-infected targets by adaptive NKG2C^+^ NK cells (Della Chiesa et al.). HCMV infection also contributes to age-associated changes in NK cells. In particular, CMV infection and age induce significant changes in the expression of certain markers including CD300a, CD161, T-bet, and Eomes in the CD57^+^ NK cell subset (Lopez-Sejas et al.). Interestingly, HCMV infection may also favor the generation of a recently identified fully mature NK cell subset, characterized by the CD56^dim^KIR^+^ LIR-1^+^NKG2A^−^ CD57^+^ phenotype, by a marked downregulation of NCRs and by the unexpected expression of the inhibitory PD-1 immune checkpoint. PD-1^+^ NK cells are also present, and in higher proportions as compared to PB of healthy donors, in both PB and ascitic fluids of ovarian-carcinoma patients suggesting their possible induction/enrichment in the microenvironment associated to the tumor. This phenotype correlates with an impaired NK cell activity toward PD-L^+^ tumor cells that can be partially restored by antibody-mediated disruption of PD-1/PD-L interaction. Importantly, the simultaneous blockade of PD-1 and KIR could amplify the NK-mediated anti-tumor activity. Due to the ineffective anti-tumor functions of this PD-1^+^ NK cell subset, it will be important to carefully evaluate the requirements that can lead to the generation of these cells in health and disease and to understand its role, in particular in patients with advanced cancers (Della Chiesa et al.).

Similar to HCMV infection, Epstein-Barr virus (EBV) infection may change the composition of NK cells. “Early-differentiated NK cells that expand during EBV infection might directly recognize lytically EBV replicating targets, while the terminally differentiated NK cells in HCMV-infected individuals mainly promote ADCC” (Chijioke et al.). Finally, the role of human NK cells in herpesviral control is accentuated by inherent genetic defects of immunity that lead to NK cell aberrations a majority of which have impact upon specific NK cell subsets (Mace and Orange). There is still much to be learned from these rare individuals with regard to specific NK cell populations and their role in human host defense.

## NK Cells and Tumors

Natural killer cell-mediated ADCC plays an important role not only in the control of infections but also of tumors and downregulation of CD16 expression on activated NK cells may limit or regulate this response. In this context, in has been demonstrated that CD56^dim^ CD57^+^ NK cells are particularly prone to losing CD16 after influenza vaccination. This event supports a role for CD16 in early activation of NK cells after vaccination and for CD16 downregulation as a means to modulate NK cell responses and maintain immune homeostasis of both antibody and T cell-dependent pathways (Goodier et al.).

Several data indicate that the microenvironment, the cytokine milieu, and genetic factors in cancer patients can exert a strong influence on NK cell receptor expression and functional potential (Carrega and Ferlazzo). Thus, the phenotype of the NK cell subpopulation associated with cancer may vary according to the specific kind of tumor and its anatomical location. In this context, chemokines/chemokine receptors play a critical role in the regulation of the distribution of NK cell subpopulations in the various tissues. Remarkably in different tumor types (including lung cancer and melanoma), both migration and homing of NK cells may be altered and even reversed. For example, NK cells present in the tumor microenvironment are often enriched in CD56^bright^CD16^neg/dim^ NK cells (Carrega and Ferlazzo); in contrast, in melanoma metastatic lymph nodes, it is possible to detect the expansion of an unusual subset characterized by a CD56^dim^ CD69^+^CCR7^+^ KIR^+^ phenotype (Cristiani et al.; Pesce et al.). A likely explanation of these events is that the release of different types of chemokines by cells of the tumor microenvironment, or the acquisition of different/new chemokine receptors by NK cells, can lead to an altered recruitment of the NK cell subpopulations.

## Translating Basic NK Cell Biology into the Clinics

Natural killer cells may display alloreactive potential in case of mismatch between recipient inhibitory KIRs and graft HLA class I molecules. Several studies have addressed the impact of this variable in the context of hematopoietic stem cell (HSC) and solid organ transplantation (including kidney transplant). In this context, several lines of evidence support that “adaptive” NKG2C^+^ NK cells may contribute to control viral infection in HSC as well as in kidney transplant recipients. On the other hand, increasing evidence supports that alloantibody-mediated NK cell activation *via* FcγRIIIA (CD16) may contribute to rejection in kidney transplant recipients. Further studies integrating phenotypic, functional, and genetic analysis of NK cells should provide valuable insights on the pathogenesis of solid organ transplant complications (Lopez-Botet et al.; Legris et al.). Importantly, in addition to NK cells, ILCs may also represent important players in the early phases after transplantation (Vacca et al.).

A major goal of cancer immunotherapy based on the use of NK cells is to reverse the tumor-induced NK cell impairment observed in cancer patients and to amplify NK cell effector functions. Therapies involving NK cells may either activate endogenous NK cells or involve transfer of exogenous pre-activated NK cells. In order to optimally harness NK cells in the next generation of NK cell-based vaccines and therapeutics single-cell studies of the variety of antigens that can lead to NK cell diversification, the definition of the significance of the human NK repertoire in the context of viral infection and tumors will be extremely important (Strauss-Albee and Blish). Many NK cell-based immunotherapies have been developed over the last decades, with allogeneic or autologous NK cells. In 2005, haploidentical NK cells were administered in a non-transplantation setting and resulted in a substantial improvement of patient clinical outcome and a substantive number of trials have followed. More recently, *in vivo* targeting of NK cells with antibodies was investigated: IPH2101 is a first-in-class anti-KIR antibody that blocks inhibitory KIR–ligand interactions, leading to restoration of NK cell functions. A phase II trial in acute myeloid leukemia elderly patients in first CR1 (NCT01687387) is in progress and several other trials are ongoing in different cancers alone or in combination with other treatments. The future introduction of a first-in-class anti-NKG2A blocking antibody (IPH2201) will also provide a novel strategy to enhance tumor cell recognition (Rey et al.; Chabannon et al.). Remarkably, the recent discovery that a subset of NK cells may express PD-1 open prospects for extending the potential of cancer immunotherapy, by combining drugs blocking PD-1 (or its major ligand PD-L1), with additional antibodies against immune checkpoints (KIR and NKG2A) expressed on this important innate effector cells (Della Chiesa et al.).

Natural killer cells can also be adoptively transferred following solid modifications in order to license them with new or reinforce functions and ensure their controlled persistence and activity in the recipient. In this context, it is crucial to analyze NK cell features in tumor patients in order to indicate a timeline when NK-mediated therapies or other immunotherapies could be performed (Rey et al.). Ongoing developments for innovative cellular therapies suggest that such progress could result in wider clinical applications in the next future (Chabannon et al.). We believe that a deeper investigation into the impact of both conventional (e.g., chemotherapy) and new therapies (e.g., anti-immune checkpoints antibodies) on NK cell homeostasis in cancer patients is now required.

A novel strategy to improve NK cell-mediated immunosurveillance is to enable a proper NK cell migration to target tissues (including tumor sites) by promoting the expression of certain chemoattractant receptors on NK cells to be used for adoptive immunotherapy. In this context, NK cells engineered *ex vivo* to express chemokine receptors by gene transfer or by trogocytosis are under investigation for their better tissue homing and function (Pesce et al.; Bernardini et al.). For example, the acquisition of CCR7 expression by CD56^dim^ KIR^+^ NK cells may play a key role in promoting the migration of this subset to lymph nodes, where CD56^dim^ KIR^+^ NK cells may shape adaptive immune responses by leading to Th1 polarization (through the release of IFNγ and the mechanism of DC “editing”) but may also prevent GvHD and HvGD in haplo-HSCT by directly killing patient’s DCs and T cells (Pesce et al.). Thus, NK cells can affect DC fate and thereby control T cell responses (Pallmer and Oxenius).

In conclusion, we express our gratitude to all the authors who have contributed to this research topic and to the reviewers for their valuable work.

## Author Contributions

All authors listed have made a substantial, direct, and intellectual contribution to the work and approved it for publication.

## Disclaimer

The content of this publication does not necessarily reflect the views or policies of the Department of Health and Human Services, nor does the mention of trade names, commercial products, or organizations imply endorsement by the U.S. Government.

## Conflict of Interest Statement

EV is a founder and shareholder of Innate- Pharma (Marseille, France). The remaining authors declare no competing financial interests.

